# Autophagy in Osteosarcoma Cancer Stem Cells Is a Critical Process which Can Be Targeted by the Antipsychotic Drug Thioridazine

**DOI:** 10.3390/cancers12123675

**Published:** 2020-12-07

**Authors:** Olivier Camuzard, Marie-Charlotte Trojani, Sabine Santucci-Darmanin, Sophie Pagnotta, Véronique Breuil, Georges F. Carle, Valérie Pierrefite-Carle

**Affiliations:** 1 Faculté de Médecine Nice, Université Côte d’Azur, UMR E-4320 TIRO-MATOs CEA/DRF/Institut Joliot, CEDEX 2, 06107 Nice, France; camuzard.olivier@hotmail.fr (O.C.); mctroj@hotmail.fr (M.-C.T.); santucci@unice.fr (S.S.-D.); breuil.v@chu-nice.fr (V.B.); carle@unice.fr (G.F.C.); 2 Service de Chirurgie Réparatrice et de la Main, CHU de Nice, 06001 Nice, France; 3 Service de Rhumatologie, CHU de Nice, 06001 Nice, France; 4 Centre Commun de Microscopie Appliquée, Université Côte d’Azur, 06107 Nice, France; sophie.pagnotta@unice.fr

**Keywords:** autophagy, osteosarcoma, cancer stem cells, Na+/K+ ATPase, autosis

## Abstract

**Simple Summary:**

Cancer stem cells (CSCs) represent a minor population of cancer cells with stem cell-like properties and appear as a crucial target in oncology as they are the origin of relapses and resistance to current treatments. Autophagy, which allows the degradation and recycling of cellular components for survival purposes, has been shown to be upregulated in some CSCs, participating in the resistance of these cells. The aim of our study was to analyze the autophagy level and the consequences of targeting this process in osteosarcoma CSCs. Our results indicate that autophagy is a critical process in osteosarcoma CSCs and that targeting this pathway allows to switch their fate from survival to death.

**Abstract:**

Cancer stem cells (CSCs) represent a minor population of cancer cells with stem cell-like properties which are able to fuel tumor growth and resist conventional treatments. Autophagy has been described to be upregulated in some CSCs and to play a crucial role by maintaining stem features and promoting resistance to both hostile microenvironments and treatments. Osteosarcoma (OS) is an aggressive bone cancer which mainly affects children and adolescents and autophagy in OS CSCs has been poorly studied. However, this is a very interesting case because autophagy is often deregulated in this cancer. In the present work, we used two OS cell lines showing different autophagy capacities to isolate CSC-enriched populations and to analyze the autophagy in basal and nutrient-deprived conditions. Our results indicate that autophagy is more efficient in CSCs populations compared to the parental cell lines, suggesting that autophagy is a critical process in OS CSCs. We also showed that the antipsychotic drug thioridazine is able to stimulate, and then impair autophagy in both CSC-enriched populations, leading to autosis, a cell death mediated by the Na+/K^+^ ATPase pump and triggered by dysregulated accumulation of autophagosomes. Taken together, our results indicate that autophagy is very active in OS CSCs and that targeting this pathway to switch their fate from survival to death could provide a novel strategy to eradicate these cells in osteosarcoma.

## 1. Introduction

Osteosarcoma (OS) is a bone cancer of mesenchymal origin which mainly affects children and adolescents [1]. Approximatively 15–20% of patients exhibit a metastatic OS at diagnosis and 25–50% will develop metastasis [2]. The overall 5-year survival rate of patients with metastatic OS is about 20% [3]. Surgery combined with chemotherapy constitutes the standard treatment but developing new therapeutic options to treat the metastatic form of this disease remains a challenge. 

Like in many other tumors, a minor population of cancer cells with stem cell-like properties called cancer stem cells (CSCs) has been identified in OS [4]. This cell population, which expresses embryonic stem cell-associated transcription factors such as Sox-2, Oct-4, Nanog, can divide asymmetrically and has the ability to self-renew [4,5,6,7,8]. It has been suggested that tumor relapses and metastasis could be due to the presence of CSCs, which may stay dormant for long periods of time, are able to fuel tumor growth and are generally spared by conventional treatments [9]. Thus, developing new strategies to target CSCs has appeared as an urgent need.

Autophagy is the major catabolic process of eukaryotic cells that degrades and recycles damaged macromolecules and organelles. During this process, the cytoplasmic material targeted to degradation is delivered to lysosomes upon sequestration within double-membraned vesicles called autophagosomes [10]. Autophagosomes and their contents are cleared upon fusing with late endosomes or lysosomes, and products of these catabolic reactions can then re-enter anabolic and/or bioenergetic metabolisms. Autophagy occurs at a low level in all cells to ensure the homeostatic turnover of long-lived proteins and organelles [10]. Autophagy is also upregulated under stressful conditions, such as starvation or hypoxia, for survival purposes [10]. Although the main goal of autophagy is survival, autophagy can also induce cell death [11]. 

In the context of cancer, autophagy can repress the initial steps of carcinogenesis through several mechanisms such as oxidative stress reduction, but this cellular process can also facilitate the development of established tumors, essentially by promoting survival [12]. Regarding CSCs, autophagy was shown to be involved in the maintenance of stem cell features [13] and pluripotency factors [14], in CSC tumorigenicity [15] and in resistance to both hostile microenvironments and treatments [16]. As a consequence, autophagy appears to be upregulated in some CSCs such as in breast [15] or ovarian cancers [17]. 

In OS, autophagy is often deregulated [18]. Indeed, among the tumor suppressor genes inactivated in OS, several such as RB1 or PTEN are positive regulators of autophagy [18]. In addition, several oncogenes activated in OS, such as IGF2, also regulate autophagy [18]. Moreover, several studies have demonstrated overactivation of mTOR in OS, suggesting a potential inhibition of autophagy [19,20]. Finally, it was recently demonstrated that loss of TP53 and RB1, the most frequently mutated genes in OS [3], induces downregulation of several autophagy genes, resulting in a decreased autophagic flux [21]. Interestingly, several OS cell lines exhibit a high basal LC3-II level [unpublished results] that could result from autophagic flux attenuation, as LC3-II is both an autophagy marker and substrate.

Regarding OS CSCs, the role of autophagy has been poorly studied. Zhang et al. used the mesenchymal CD271 marker to isolate OS cells exhibiting stem cell properties [22]. CD271+ cells isolated from OS cell lines were shown to have a higher autophagic activity than CD271- cells under hypoxia and low nutrient conditions. In addition, autophagy was shown to participate in CD271+ cell stemness and contribute to tumorigenicity and drug resistance in vitro and in vivo [22]. 

In the present study, we chose autophagy-competent and autophagy-inefficient OS cell lines to isolate CSC-enriched populations and to compare the autophagic process in basal and nutrient-deprived conditions. We also demonstrated that thioridazine (TZ), an antipsychotic drug known to target CSCs and to interfere with autophagy, is able to engage these cells in a specific death pathway called autosis. 

## 2. Results

### 2.1. Selection of OS Cell Lines with Different Autophagic Profiles

**Figure 1:**
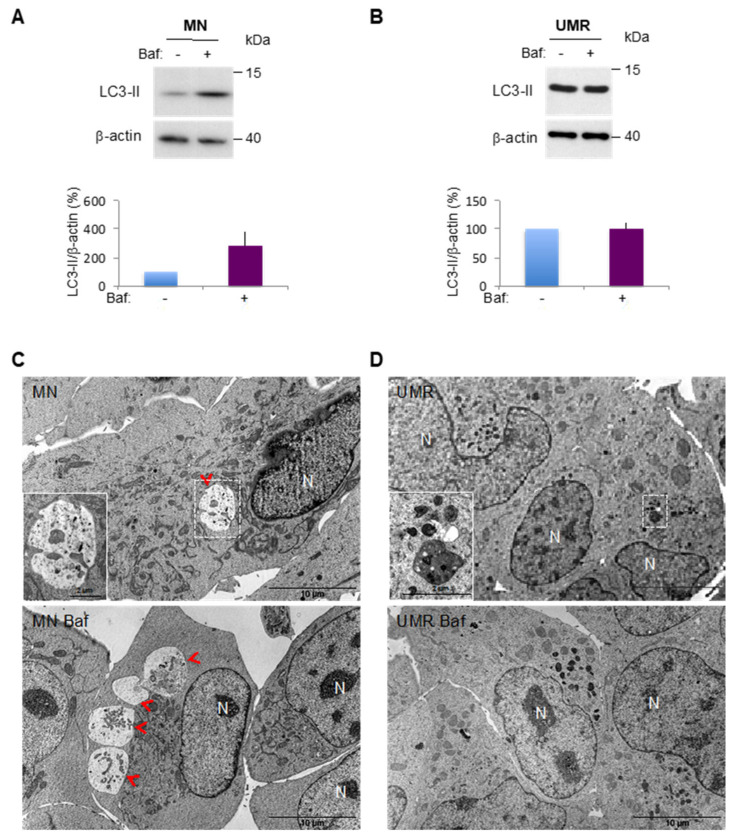
Selection of Osteosarcoma (OS) cell lines with different autophagic profiles. (**A**,**B**) LC3-II expression was analyzed by Western blot in the MNNG/HOS (MN) and UMR-106 (UMR) cell lines, in the absence (−) or presence (+) of Bafilomycin A1 (Baf) to analyze the autophagic flux. LC3-II to β-actin relative expression levels are presented. The results are normalized relative to the control in the absence of Baf. Histograms represent the mean ± SEM of three independent experiments. (**C**,**D**) TEM analysis of the MNNG/HOS (MN) and UMR-106 (UMR) cell lines. Lower panels: Baf was added to block the autophagic flux one hour prior to sample fixation. Autophagic vesicles magnification is presented in the white square. Red arrowhead: autophagosome. N: nucleus.

For the isolation of CSC-enriched populations, we chose two OS cell lines exhibiting different profiles of autophagic activity, the MNNG/HOS (MN) human cell line and the UMR-106 (UMR) rat cell line. Basal autophagy of these cells was analyzed by western blot focusing on the expression of the essential autophagy protein microtubule-associated protein 1 light chain 3 protein (LC3-II), which is considered as an autophagosome marker [23]. However, autophagy is a dynamic mechanism in which vesicles are formed and rapidly degraded by fusion with the lysosome, in a process called “autophagic flux”. Thus, we monitored LC3-II turnover by using the lysosomal proton pump inhibitor Bafilomycin-A1 (Baf) to clamp the LC3-II autophagosome degradation [23]. Therefore, the autophagic flux can be evaluated by comparing the LC3-II level in the absence and in the presence of Baf, which corresponds to the amount of LC3-II delivered to the lysosome for degradation. As shown in Figure 1A, in the MN cell line, Baf addition results in an increased LC3-II signal (281% in the presence of Baf vs. 100% in its absence), suggesting a dynamic autophagic flux. Conversely, Baf addition in the UMR cell line doesn’t increase the LC3-II signal (99.85% in the presence of Baf vs. 100% in its absence), indicating a virtually null autophagic flux (Figure 1B). To illustrate these results, we next performed a transmission electron microscopy (TEM) analysis of the cells allowing us to visualize the autophagic vesicles. As shown in Figure 1C, the MN cell line presented large autophagosomes which were more numerous in the presence of Baf, indicating a dynamic autophagic flux. In the presence or in the absence of Baf, the UMR cell line essentially exhibited dark vesicles with incompletely digested material evoking autolysosomes, which is consistent with a blocked autophagic flux (Figure 1D).

### 2.2. Isolation and Characterization of CSC-Enriched Populations from OS Cell Lines

**Figure 2:**
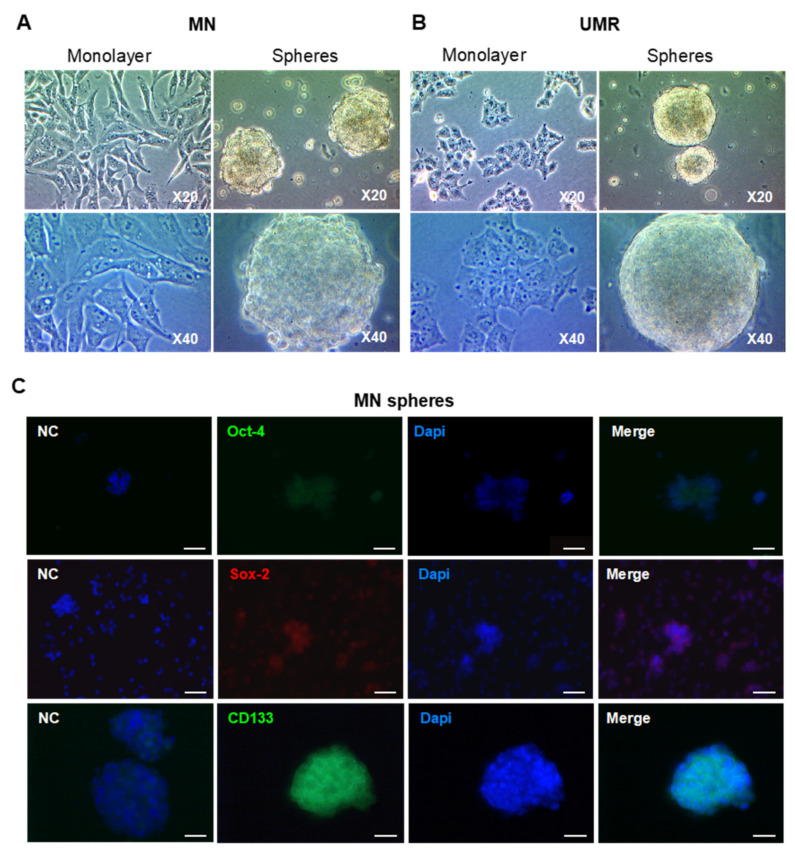

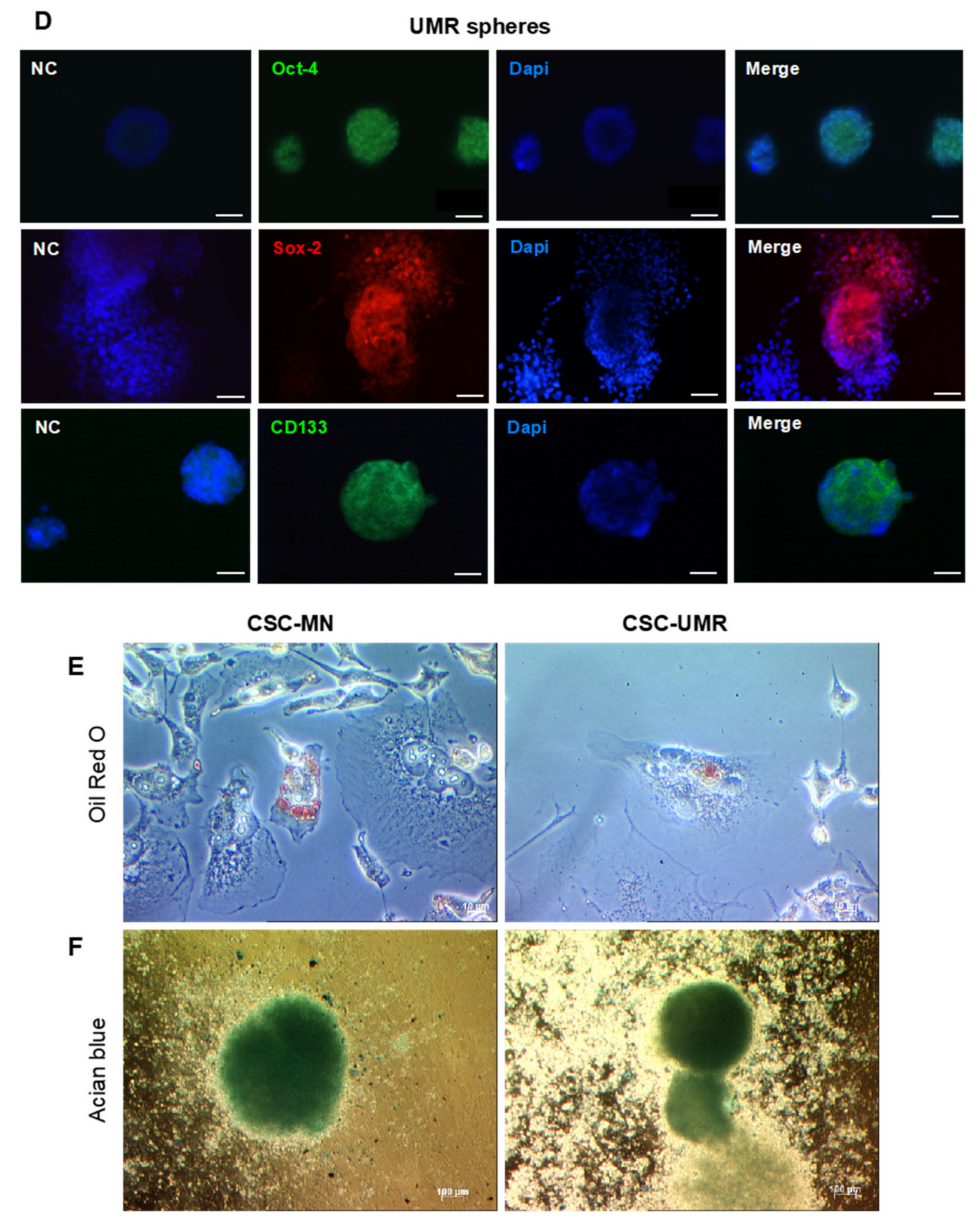
Isolation and characteristics of Cancer stem cell (CSC)-enriched populations from OS cell lines. (**A**,**B**) Representative pictures of human MN and rat UMR cells grown as monolayers or spheres at ×20 and ×40 magnifications. (**C**,**D**) Immunofluorescent labeling of Oct-4 (green), Sox-2 (red) and CD133 (green) in MN and UMR spheres. Nuclei are stained with Dapi (blue). Samples stained with secondary antibodies alone served as negative controls (NC). Magnification ×20, scale bar: 50 µm. (**E,F**) Differentiation towards adipogenic (oil red O staining, magnification ×40) and chondrogenic (alcian blue staining, magnification ×5) lineages following culture of CSC-MN and CSC-UMR in specific differentiating conditions.

We next isolated CSC-enriched populations (CSCs) from the two OS cell lines by culture in suspension in serum-free medium. After seven to ten days, both OS cell lines were able to form spheres that proliferate, suggesting a self-renewal ability (Figure 2A,B). After sphere dissociation and re-seeding at low density, the cells were able to reform spheres repeatedly. As CSC self-renewal and pluripotency is associated with the expression of specific transcription factors, we next analyzed the expression of Oct-4 and Sox-2, two proteins known to be expressed in OS CSCs [4,5,6,7]. While the CSC-UMR highly express Oct-4, a poor expression was observed in the CSC-MN. Nevertheless, both CSC-enriched populations express the Sox-2 transcription factor (Figure 2C,D) and the classical CSC marker CD133 (Figure 2C,D).

As OS CSCs were described to exhibit some mesenchymal stem cell (MSC) characteristics, we further analyzed their ability to differentiate towards adipogenic and chondrogenic lineage following culture in specific differentiating conditions. As shown in Figure 2E, visible Oil Red O-positive droplets containing-cells were observed from seven days in adipogenic differentiation medium. Similarly, differentiation towards the chondrogenic lineage was observed after 11 days of culture in chondrogenic conditions (Figure 2F). Such differentiation capacities were not observed in the parental UMR and MN cell lines (Figure S1). Taken together, these results suggest that OS cell culture in stem cell-specific conditions allows the isolation of CSC-enriched populations exhibiting some stem cell markers as well as differentiation potential in the chondrogenic and adipogenic lineages.

### 2.3. Comparison of Basal and Stress-Induced Autophagy in CSC-Enriched Populations and OS Parental Cells

**Figure 3:**
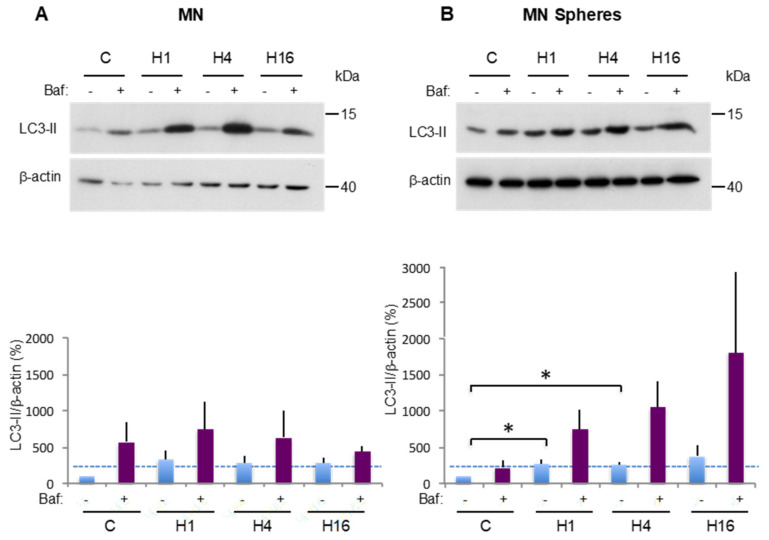

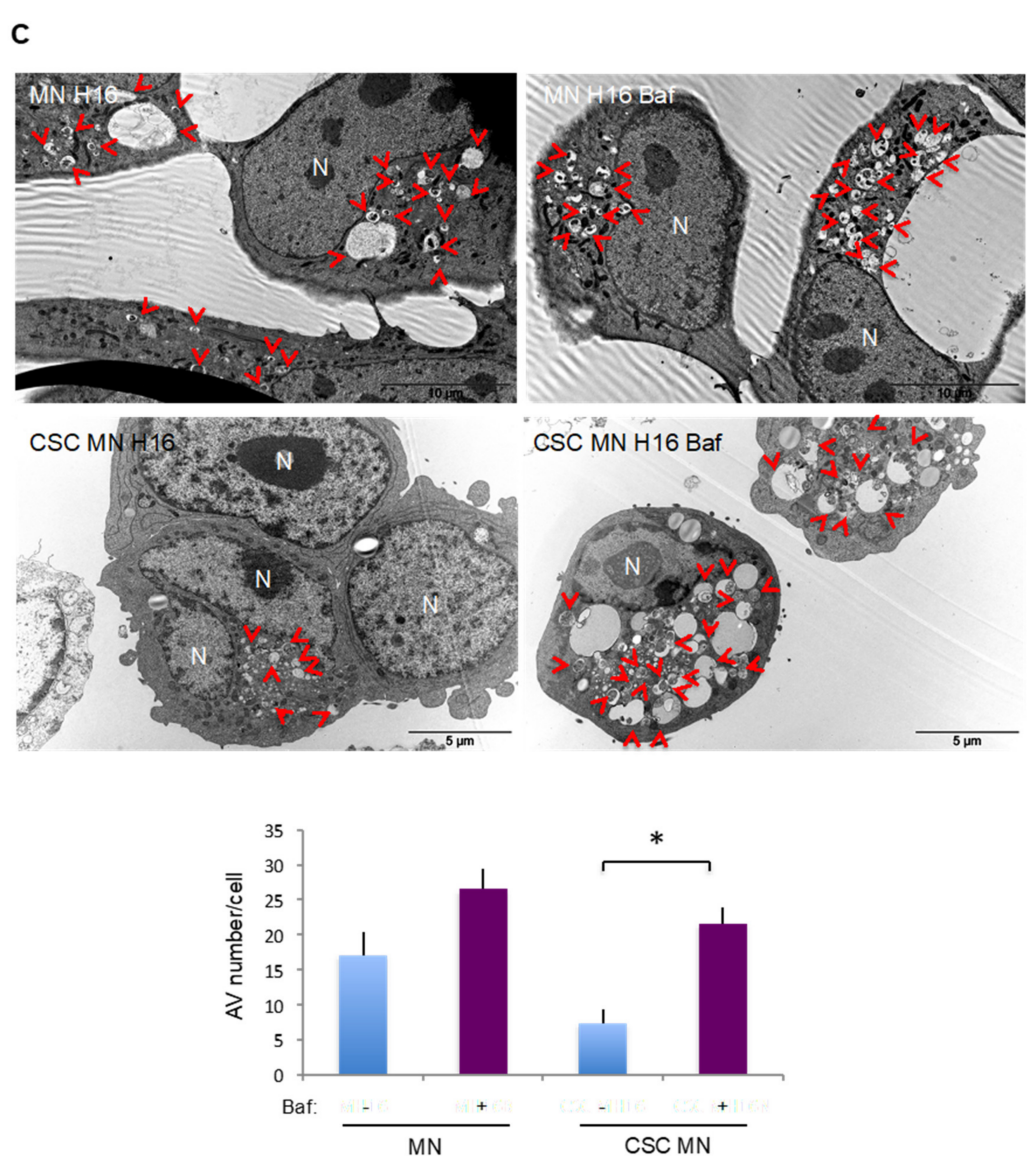
Comparison of basal and stress-induced autophagy in CSC MN-enriched population and MN parental cells. (**A**,**B**) LC3-II expression was analyzed by Western blot in the MN cell line and in the corresponding CSC-enriched population, in complete medium (**C**) and at 1 (H1), 4 (H4) and 16 (H16) hours after HBSS incubation. One hour prior to protein extraction, Baf was added (+) or not (−) to block the autophagic flux. LC3-II to β-actin relative expression levels are presented. The results are normalized relative to the control in the absence of Baf. Histograms represent the mean ± SEM of three independent experiments. The dashed line points to the LC3-II level in the control condition (**C**) TEM analysis of the MN cell line and in the corresponding CSC-enriched population after 16 h of HBSS treatment (H16). Histograms represent the mean ± SEM of the number of autophagic vesicles (AV) per cell. Red arrowhead: autophagic vesicle. N: nucleus. NS: not significant. *: *p* < 0.05.

We first compared basal and stress-induced autophagy in the MN cell line and the corresponding CSC-enriched spheres. To this aim, the cells were incubated in complete or nutrient-deprived medium (Hank’s balanced salt solution (HBSS)) for various times. The results obtained for the MN parental cell line are presented in Figure 3A. In complete medium (C), a discrete LC3-II signal is observed, with an increase in the presence of Baf, suggesting an efficient autophagic flux in basal conditions, as previously observed. After 1 h (H1) or 4 h (H4) in HBSS, an increase in LC3-II signal intensity is observed compared to the complete medium, although the results were not statistically significant. This signal was further increased by the addition of Baf, indicating an efficient autophagic flux. Maximum stimulation of autophagy was observed after 1 h in HBSS (324% in H1 vs. 100% in C). After 16 h in HBSS, an attenuation of the autophagic flux was observed. These results are strengthened by TEM analysis (Figure S2A). 

The results obtained for the MN spheres are presented in Figure 3B. In basal conditions and after 1 h or 4 h in HBSS, the results are almost comparable to those obtained for the MN parental cell line, but the LC3-II increase in H1 vs. C and H4 vs. C is statistically significant. These results are also illustrated by representative TEM pictures of each condition (Figure S2B). An interesting difference can be observed for the H16 condition, where Baf addition induces a very important accumulation of autophagosomes (1805% in H16 + Baf vs. 374% in H16) (Figure 3B), indicating a rapid autophagic flux in MN spheres, whereas it appears to be modest in the parental MN cell line (437% in H16 + Baf vs. 283% in H16) (Figure 3A). To reinforce this result, we counted the autophagosome number in the H16 condition in TEM pictures of MN cells and corresponding spheres (Figure 3C). The number of autophagosomes observed in MN cells in the absence and in the presence of Baf was not significantly different, while there was a significant 3-fold increase in autophagosome number in CSCs in the presence of Baf. Hence, although a flux attenuation is observed in parental cells, it appears to be still active in spheres. Taken together, these results suggest that while autophagy is dynamic in MN cells, the corresponding CSCs appear to respond even better to sustained, deprivation-dependent stimulation.

**Figure 4:**
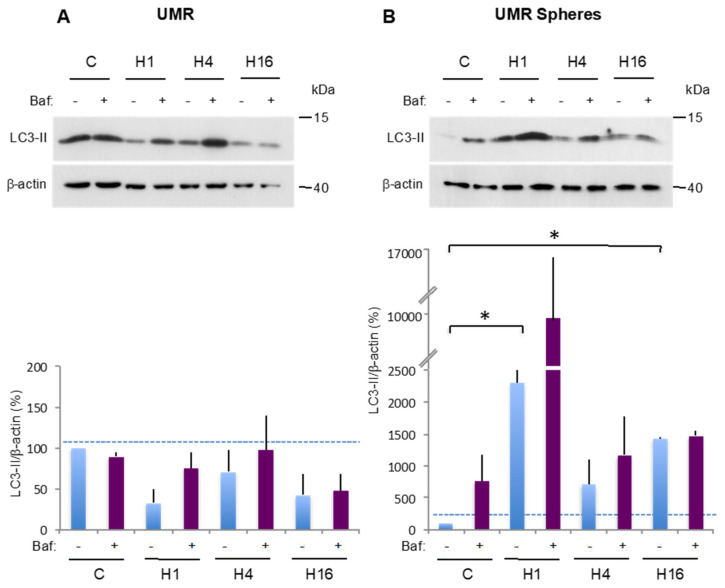

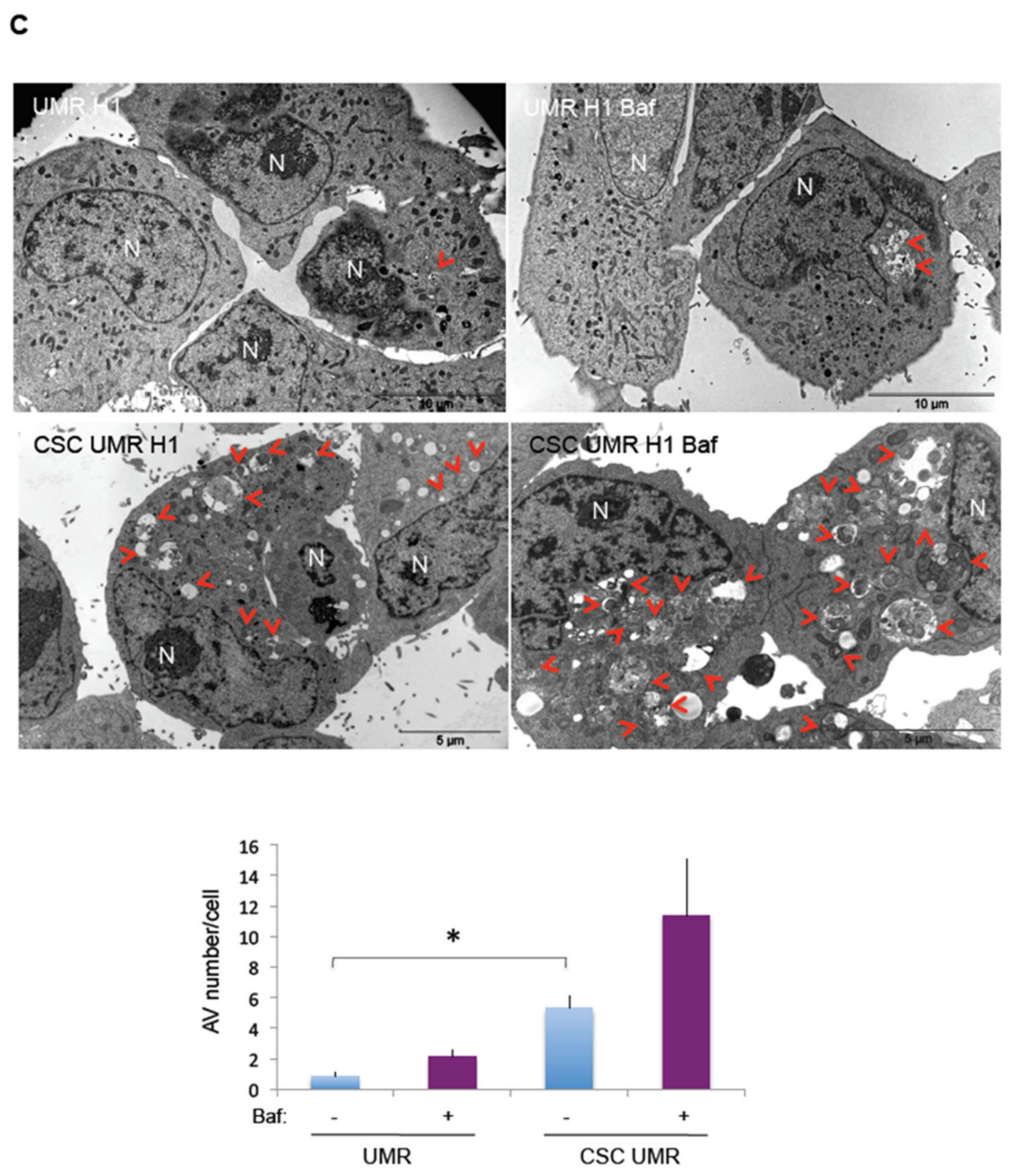
Comparison of basal and stress-induced autophagy in CSC UMR-enriched population and UMR parental cells. (**A**,**B**) LC3-II expression was analyzed by Western blot in the UMR cell line and in the corresponding CSC-enriched population, in complete medium (**C**) and at 1 (H1), 4 (H4) and 16 (H16) hours after HBSS incubation. One hour prior to protein extraction, Baf was added (+) or not (−) to block the autophagic flux. LC3-II to β-actin relative expression levels are presented. The results are normalized relative to the control in the absence of Baf. Histograms represent the mean ± SEM of three independent experiments. The dashed line points to the LC3-II level in the control condition (**C**) TEM analysis of the UMR cell line and the corresponding CSC-enriched population after 1 h of HBSS treatment (H1). Histograms represent the mean ± SEM of the number of autophagic vesicles (AV) per cell. Red arrowhead: autophagic vesicle. N: nucleus. *: *p* < 0.05.

We then performed the same analysis in the UMR cell line and the corresponding CSC-enriched UMR spheres. Figure 4A shows a representative western blot experiment performed in the UMR parental cell line. In complete medium, a strong LC3-II signal was observed whose intensity did not increase after Baf addition, suggesting a null autophagic flux in basal conditions, as previously observed. After 1 h or 4 h in HBSS, the presence of Baf induced a slight increase in the LC3-II signal, suggesting a “restart” of the autophagic flux. After 16 h in HBSS (H16), the autophagic flux was basically blocked, as seen in the control condition. Collectively, these data indicate that the UMR cell line exhibits a poor autophagy response to starvation, which is consistent with the high mortality rate observed after 16 h in HBSS (Figure S3). These results are illustrated by representative TEM pictures presented in Figure S4A. 

The results obtained for UMR spheres are presented in Figure 4B. In complete medium, Baf addition induced a strong increase in the LC3-II signal (765% in C + Baf vs. 100% in C), indicating a high autophagic flux. A significant stimulation of autophagy was observed after 1 h in HBSS (2304% in H1 vs. 100% in C). Moreover, in this condition, Baf addition induced a very important autophagosome accumulation, indicating a very rapid flux. After 4 h in HBSS, autophagy decreased but remained important (707% in H4 vs. 100% in C). After 16 h in HBSS, although there is a significant increase in LC3-II signal compared to the control condition, a flux inhibition is observed (1465% in H16 + Baf vs. 1431% in H16), like in the parental cell line. TEM analysis confirmed these data showing a dynamic autophagic flux except for H16 (Figure S4B). The marked difference observed between parental UMR cells and the corresponding spheres after 1 h in HBSS is illustrated by representative TEM pictures (Figure 4C). The number of autophagic vesicles per cell was significantly higher in CSCs compared to the parental UMR cells. These results indicate that autophagy is not dynamic in UMR cells whereas this process seems to be very reactive in the corresponding CSCs. 

Altogether, these results indicate that autophagy is more efficient over time in CSCs obtained from an autophagy-competent cell line (MN), and that it is upregulated in CSCs obtained from a cell line which is poorly efficient in autophagy (UMR), suggesting that this process is a critical process in OS CSCs.

### 2.4. The Antipsychotic Drug Thioridazine Induces an Autophagy Stimulation Followed by an Inhibition of the Autophagic Flux in OS CSC-Enriched Populations 

**Figure 5:**
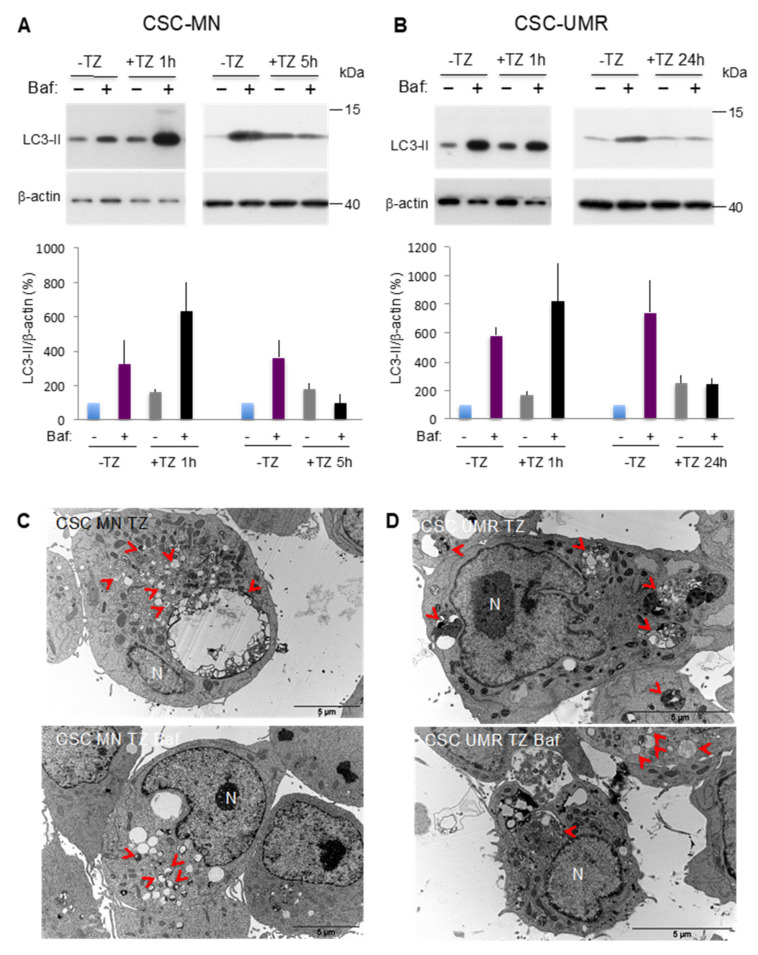
Thioridazine (TZ) modulates the autophagic pathway ultimately leading to autophagic flux impairment in OS CSC-enriched populations. (**A**,**B**) Western blot analysis of the LC3-II protein expression performed in the absence (-) or in the presence (+) of Baf and TZ at sub-toxic concentration (8 µM) for a short (1 h) or longer (5 h or 24 h for the MN or the UMR cell line, respectively) time. LC3-II to β-actin relative levels are presented. Histograms represent the mean ± SEM of three independent experiments. (**C**,**D**) Representative TEM pictures of CSC UMR and CSC MN treated with TZ at 8 µM in the absence or presence of Baf. Red arrowhead: autophagic vesicle. N: nucleus.

As autophagy appears to be a critical process in OS CSCs, we next searched for a candidate drug that could interfere with this process and induce cytotoxicity. Thioridazine (TZ) is a dopamine receptor D2 antagonist used for many years as an antipsychotic drug. More recently, it has been identified from libraries of known compounds as a CSC-targeting drug in a neoplasic pluripotent stem cell model [24]. Since then, TZ was described to induce cell death in several CSC models [25]. In addition, TZ was shown either to stimulate [26,27,28,29] or to inhibit late-stage autophagy [30]. We first analyzed autophagy in both CSC-enriched populations treated by a sub-toxic TZ concentration (8 µM) for different times. Our results indicate that TZ stimulates autophagy after a 1 h-treatment and then blocks the autophagic flux in both populations after longer treatments (5 h in the MN cell line and 24 h in the UMR cell line), as Baf is no longer able to increase the LC3-II signal in the presence of TZ (Figure 5A and B). To confirm these results, we analyzed by TEM both CSC-enriched populations treated by TZ (8 µM 5 h for CSC MN and 24 h for CSC UMR). In CSC MN, TZ treatment leads to the accumulation of numerous autophagic vesicles, in the absence and in the presence of Baf, illustrating inhibition of the autophagic flux (Figure 5C). In CSC UMR, we observed large autophagic vesicles, which can be consistent with a maturation defect such as lysosome fusion, both in the absence and in the presence of Baf (Figure 5D).

### 2.5. Thioridazine Induces Autosis in OS CSC-Enriched Populations 

**Figure 6:**
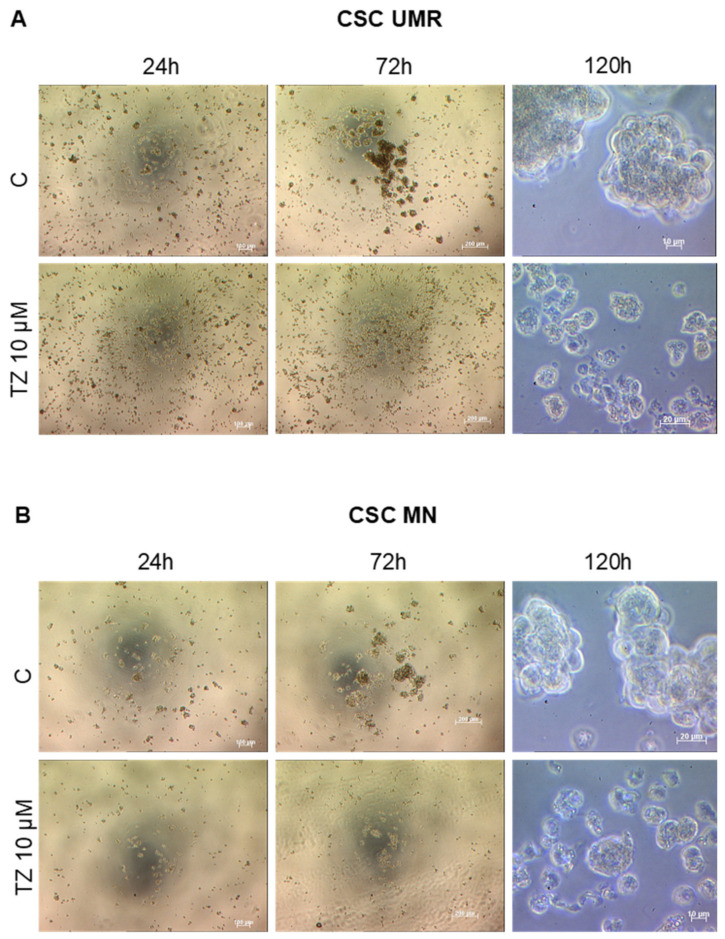

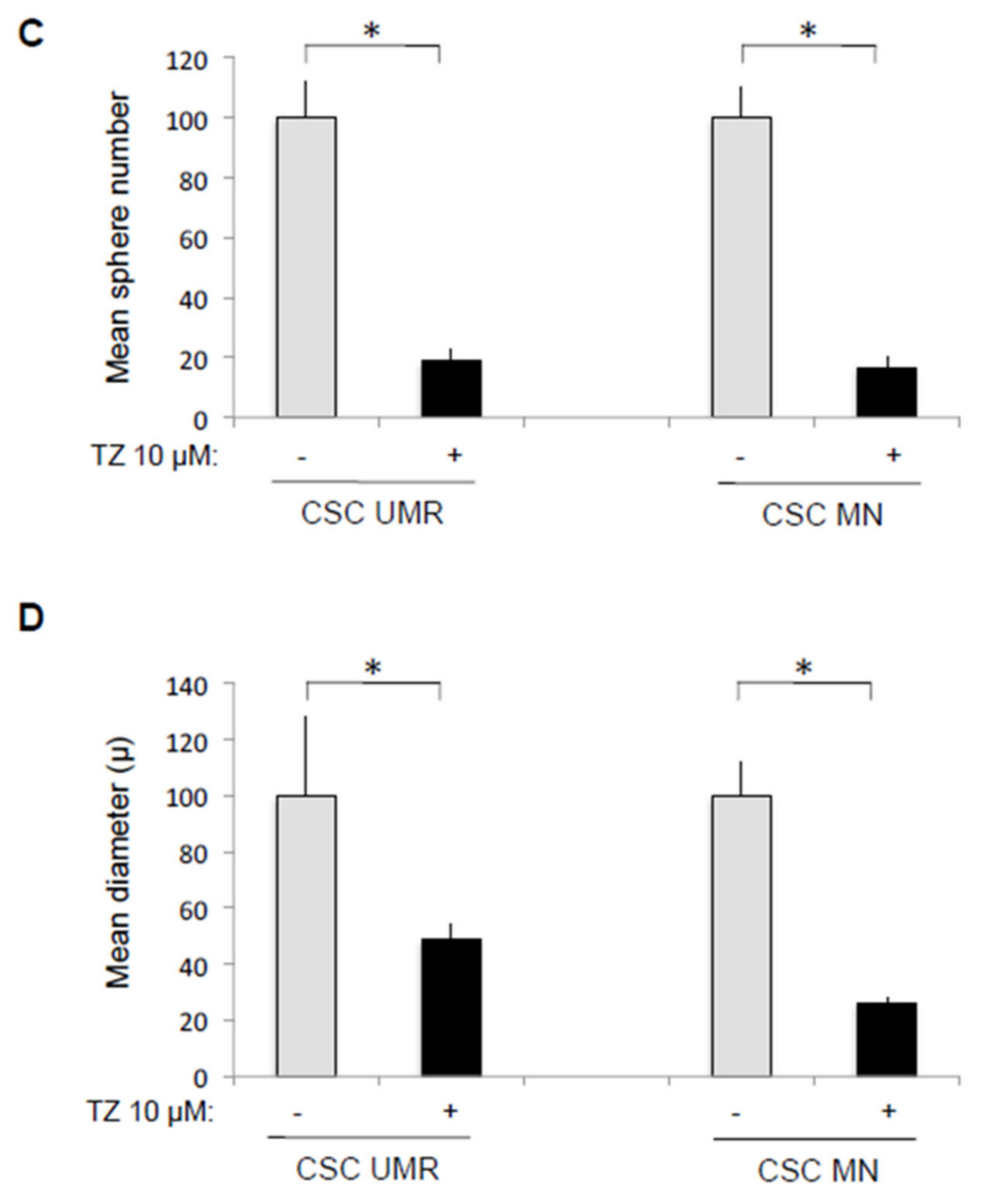
TZ inhibiting OS sphere formation. (**A**,**B**) Sphere formation in the absence (Control, C) or in the presence of TZ at 10 µM. Representative photographs at 24, 72 h (magnification ×5) and 120 h (magnification ×40). (**C**) Mean sphere number at 120 h normalized to control in each condition. (**D**) Mean diameter at 120 h normalized to control in each condition. * *p* < 0.05 (Student *t* test).

Next, we analyzed the cytotoxicity of TZ in UMR and MN CSC-enriched populations. Based on literature and previous experiments (data not shown), TZ treatment for 24 h at 10 µM kills the majority of cells in both CSC-enriched populations. To test whether some cells could recover after this treatment, we analyzed sphere development during the five days after seeding in the absence or in the presence of TZ 10 µM (or 20 µM, data not shown). As shown in Figure 6A,B, we observed that TZ inhibited large sphere formation in both CSC-enriched populations (Figure 6C,D).

**Figure 7:**
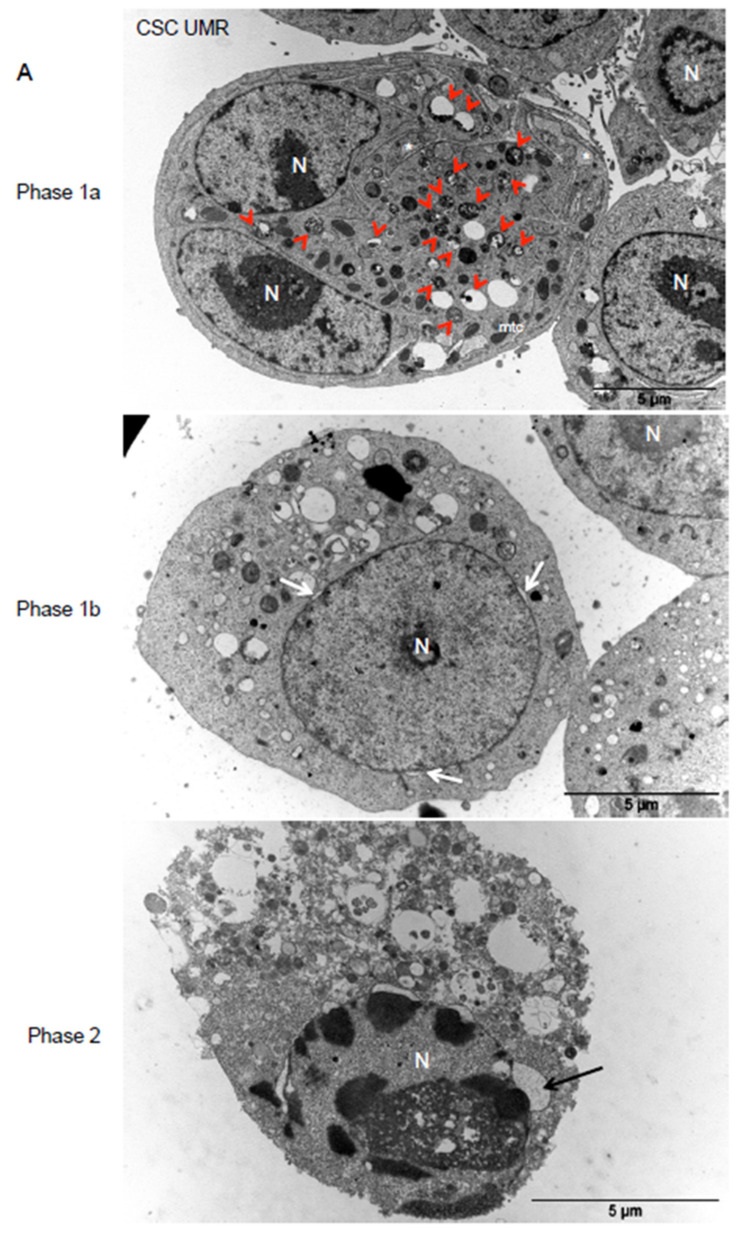

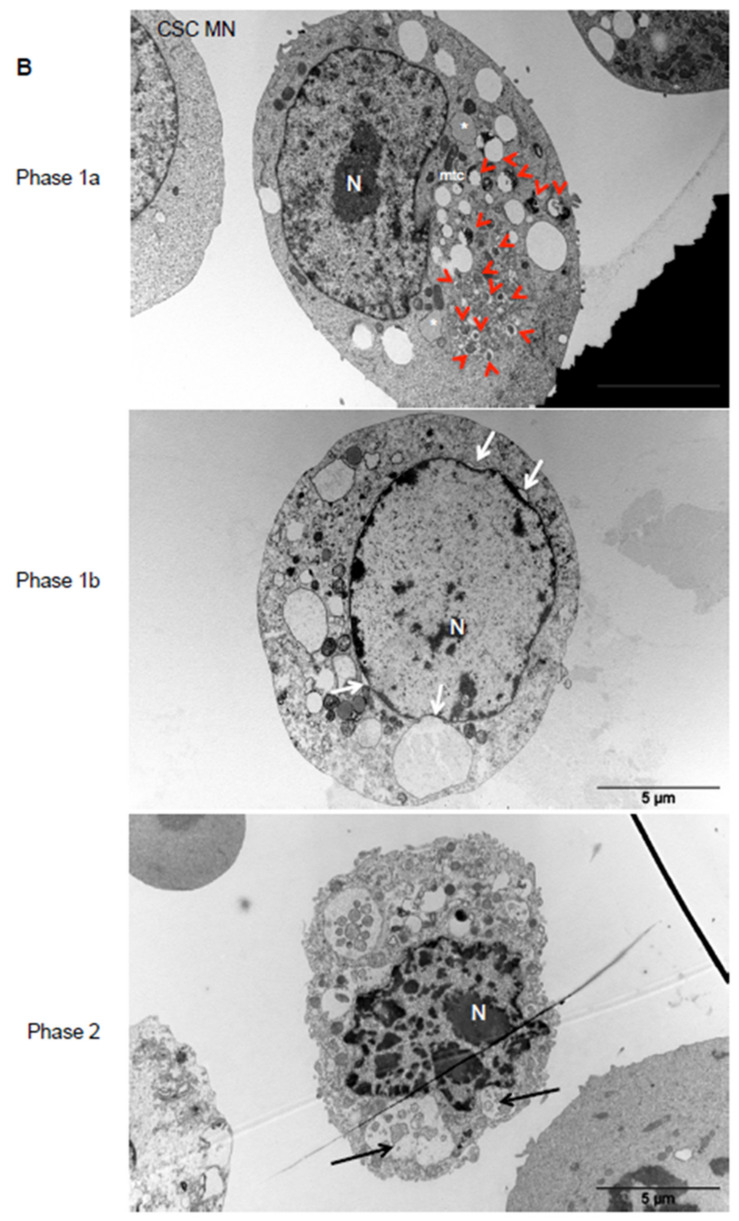
Morphological features of TZ-induced cell death evokes autosis, a Na + /K+ ATPase-dependent cell death. Representative TEM pictures of the different phases of TZ-induced cell death. UMR (**A**) and MN (**B**) CSC-enriched cells were treated with different TZ concentrations (8 or 20 µM) for various periods (3 h 30 to 24 h) and representative pictures of the different phases of autosis are shown. Autophagic vesicles (red arrowheads), electron-dense mitochondria (mtc) and endoplasmic reticulum (ER) dilatation (*) are observed in phase 1a. Local separation of the inner and outer nuclear membranes (white arrows) is shown in phase 1b. Focal ballooning of the perinuclear space (black arrows) and lysis of the plasma membrane are observed in phase 2. N: nucleus.

To further investigate cell behavior during TZ-induced cell death, we performed TEM experiments after treatment with different TZ concentrations for various periods (Figure 7A,B). We observed morphological features evoking the different phases of autosis, a cell death mediated by the Na+/K^+^ ATPase pump and triggered by dysregulated accumulation of autophagosomes [31,32,33]. In the early phase of TZ toxicity (Phase 1a), we observed the presence of numerous autophagic vesicles (or large autophagic vesicles) and empty vacuoles, electron-dense mitochondria and endoplasmic reticulum (ER) dilatation. In a second phase, perinuclear space became swollen at discrete region (Phase 1b). In the last phase, there was a focal ballooning of the perinuclear space and lysis of the plasma membrane (Phase 2). 

**Figure 8:**
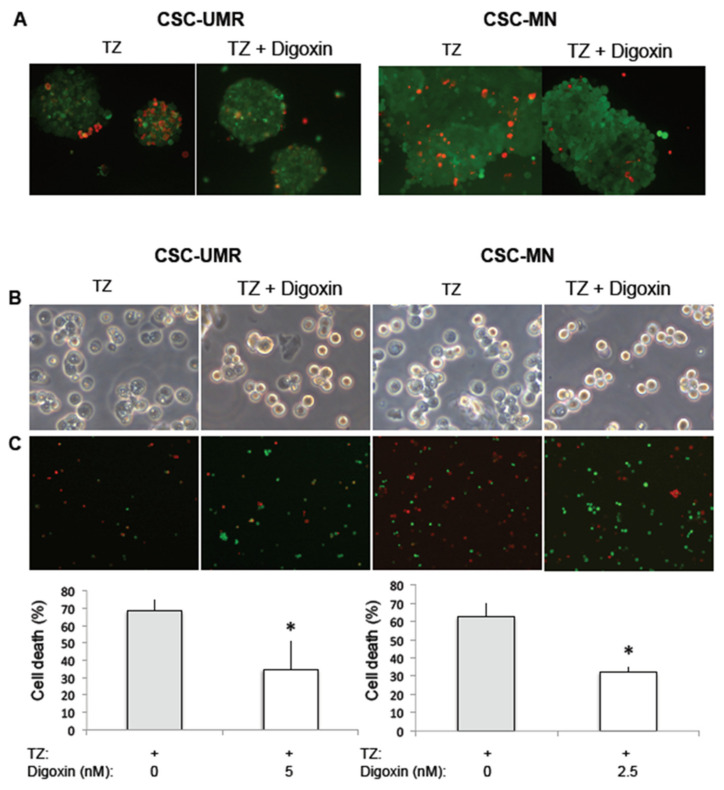
Digoxin rescues TZ-induced autosis in OS CSC-enriched populations. (**A**) The UMR or MN spheres were treated with TZ at 8 µM for 48 h, in the absence or in the presence of Digoxin 5 nM. Representative pictures of spheres analyzed using the Live/Dead® Viability/cytotoxicity Kit. Live cells are labeled in green, and dead cells display red nuclei (×10 magnification). (**B**) After sphere dissociation, the cells were treated by TZ at 30 µM for 3 h, in the absence or in the presence of Digoxin. Phase contrast light microscopy pictures of the CSC-enriched populations in the different conditions. (**C**) Cell death analysis using the Live/Dead® Viability/cytotoxicity Kit. Representative fields observed for each condition are presented (×10 magnification). Histograms represent mean cell death ± SEM obtained through the analysis of 500–600 cells per condition. *: *p* < 0.05.

In order to confirm that TZ induces autosis in OS CSC, we next determined the effect of Digoxin, a potent inhibitor of autosis [31], on TZ-induced cell death. We first performed the analysis in spheres treated by TZ, using a Live/Dead Viability/cytotoxicity test. As shown in Figure 8A, Digoxin was able to reduce the number of dead (red) cells within spheres of both populations. To quantify the effect of Digoxin, we then performed the same experiments on isolated cells after sphere dissociation. Digoxin was able to significantly decrease TZ-induced cell death in both CSC-enriched populations (Figure 8B,C), suggesting that autosis is triggered by TZ in these cells.

## 3. Discussion

CSCs represent a particularly important target in oncology as they are at the origin of relapses and resistance to current treatments. Among the survival mechanisms developed by these cells, autophagy seems to play a crucial role by maintaining stem characteristics, limiting oxidative stress, and promoting the resistance and adaptation of CSCs to their microenvironment [16]. Therefore, it seems logical that CSCs upregulate their autophagic activity compared to the differentiated tumor cells, as it has been observed in CSCs from breast [15] or ovarian [17] cancers. In the context of OS, very few studies have been performed on autophagy in CSCs. However, this is a very interesting case because autophagy is deregulated in this cancer [18], with a potential autophagic flux inhibition due in particular to TP53 and RB1 mutations [21]. Indeed, several OS cell lines exhibit a high basal LC3-II level that could result from autophagic flux attenuation, as observed in other cancer cell lines such as MDA-MB-231 [23] [unpublished results]. For this reason, we used two model OS cell lines exhibiting different autophagic profiles to compare basal and starvation-induced autophagy in these cells and their corresponding CSC-enriched populations. The CSC-enriched populations were selected by culture in specific serum-free medium and maintained as spheres which express the stem cell transcription factors Oct-4, Sox-2 and the classical CD133 CSC marker. These cells also exhibit some MSC-like differentiation potential, as previously demonstrated [6].

Regarding the autophagy-competent MN model, autophagy appears to be more robust in the CSC-enriched population as the autophagic flux is greater after 16 h in HBSS, compared to the parental cell line. For the UMR model exhibiting a poor autophagic profile, the number of autophagic vesicles and the autophagic flux are dramatically increased in the CSC-enriched population in control and H1 conditions compared to parental cells. These results indicate that autophagy is considerably increased even if CSCs are isolated from an autophagy-inefficient OS cell line. Recent publications have shed light on these findings, by showing that some stem cell transcription factors are able to upregulate the expression of autophagy genes. It was first demonstrated that Sox-2 increases ATG10 expression, resulting in autophagy stimulation [34]. Similarly, Oct-4 knockdown in CSC was shown to decrease protein and mRNA levels of ATG5, ATG7, ATG12, or LC3-II [14]. It was also shown that Nanog can activate autophagy through direct binding to the BNIP3L promoter [35] and to upregulate LC3 expression [36]. Taken together, these data suggest that autophagy is a critical process in CSCs, including in OS CSC.

In order to target this Achille’s heel of the CSCs, we next searched for a candidate drug that could interfere with autophagy and induce cytotoxicity. We selected TZ, an antipsychotic antagonist of the DRD2 dopamine receptor, which has been tested as a repositioned drug in CSCs from myeloid leukemia, glioblastoma, lung, liver, ovarian and breast cancers [25]. Apart from apoptosis, TZ was also described as an autophagy inducer in glioblastoma, ovarian, cervical and melanoma cancer cells [26,27,28,37]. Inversely, TZ was shown to inhibit late-stage autophagy by impairing fusion between autophagosomes and lysosomes in glioblastoma cells [30]. Our results can indeed reconciliate the divergent data reported in the literature, as we demonstrated that TZ treatment results in an initial autophagy induction followed by an autophagic flux inhibition. 

Next, we showed that TZ is able to inhibit sphere development, significantly reducing both sphere number and diameter in both CSC models. Using TEM, we also observed that TZ induces morphological features evoking autosis, a cell death mediated by the Na^+^/K^+^ ATPase pump and triggered by dysregulated accumulation of autophagosomes [31,33]. Autosis is a specific form of cell death identified by Levine’s group and observed in cells submitted to powerful autophagy inducers such as starvation or autophagy-inducing peptides [31]. More recently, autosis was shown to be associated with a strong autophagy stimulation followed by attenuation of the autophagic flux, upregulation of Rubicon and a marked accumulation of autophagosomes [33]. Indeed, while autosis is an autophagy-dependent cell death, this process only requires the early stages of autophagy and an autophagic flux inhibition per se doesn’t impair cell death [31,33]. Autosis is dependent on the Na^+^/K^+^ ATPase pump, and cardiac glycosides such as Digoxin, which are Na^+^/K^+^ ATPase antagonists, have been identified as autosis inhibitors in a chemical screen of 5000 compounds [31]. We showed that Digoxin was able to significantly reduce TZ-induced cell death in both CSC-enriched populations, confirming autosis induction by TZ.

To date, this cell death has only been observed in cases of hypoxia-ischemia in mice brain [31], in mouse kidneys subjected to ischemia-reperfusion [38] and during cardiac ischemia-reperfusion [33]. In these situations, autosis appears to contribute to the injury, and strategies to target autosis in these pathologies should alleviate disease severity [39]. In the context of cancer, very few studies have been carried out on autosis. To our knowledge, our study constitutes the first evidence of autosis induction in CSCs. Regarding the mechanism, increasing evidences suggest that autotic cell death could be mediated through excessive autophagosome accumulation [39]. Hence, inhibition of the autophagic flux by TZ could trigger autosis. This excessive autophagosome accumulation could contribute to autosis through depletion of intracellular membranes used to generate autophagic vesicles, as demonstrated in cardiomyocytes treated by the autophagy-inducing peptide Tat-Beclin [33,39].

In conclusion, we demonstrated that autophagy is more efficient in OS CSC-enriched populations compared to parental cell lines, with a dramatically increased autophagic flux in the UMR murine model or a more sustainable process over time in the MN human model. These results suggest that autophagy is a critical process in OS CSCs. Then, we targeted this autophagy addiction by the use of TZ, which stimulates this process and then inhibits the autophagic flux in both OS CSC-enriched populations, leading to cell death by autosis. Collectively, these data suggest that targeting autophagy in CSCs could allow a switch from survival to death, providing a novel strategy to eradicate these cells in osteosarcoma.

## 4. Materials and Methods 

### 4.1. Cell Culture

The UMR-106 (UMR) cell line corresponds to an osteogenic sarcoma induced in a rat by serial injections of ^32^P [40]. The MNNG/HOS (MN) cell line was derived from an osteosarcoma tumor present in a 13 year-old caucasian female. The cells were then treated with a carcinogenic nitrosamine [41]. The UMR and the MN cell lines were maintained in Dulbecco’s modified Eagle medium (DMEM, Lonza, Levallois-Perret, France) and DMEM-F12 (Sigma-Aldrich, St Quentin Fallavier, France) respectively, supplemented with 10% Hyclone fetal calf serum (Thermo Scientific, Villebon sur Yvette, France). Populations enriched in CSCs were cultured in ultra-low attachment flasks (Corning, Fisher Scientific, Illkirch, France) in DMEM-F12 methylcellulose 1% supplemented with 10 ng/mL basic fibroblast growth factor (bFGF), 20 ng/mL epidermal growth factor (EGF) (Peprotech, Neuilly sur Seine, France) and N2 supplement (Invitrogen, Fisher Scientific). Sphere dissociation for cell dilution was performed each week upon incubation with Accumax (Sigma-Aldrich), a mix of mild proteases, at 37°C for 10 min. The spheres were transferred at least five times before use and were dissociated two days before each experiment in order to obtain small spheres enriched in CSCs.

### 4.2. Immunofluorescence

Cells were fixed in formaldehyde 4% for 10 min at room temperature. After permeabilization in Triton X-100 0.1% and SDS 0.02% during 5 min at RT, slides were blocked in 5% nonfat milk for 20 min at room temperature. Primary antibodies for Oct-4 (Abcam, Paris, France), CD133 (Abcam) and Sox-2 (Cell Signaling Technology, St Quentin en Yvelines, France) were diluted 1:100, 1:200 and 1:50 respectively, and added to slides overnight at 4 °C in a humidified chamber. Slides were rinsed and incubated with secondary goat anti-rabbit (Oct-4 and CD133) or anti-mouse (Sox-2) Alexafluor antibodies for 1 h at room temperature. Slides were rinsed, mounted with Dapi and analyzed using a Zeiss High-throughput Live Epifluorescence Microscope (Axio Observer Z1 motorized inverted microscope).

### 4.3. Differentiation Towards Adipogenic and Chondrogenic Lineages

Cells dissociated from spheres were cultured in adipogenesis differentiation medium (Stempro adipogenesis differentiation kit, Gibco, Thermo Scientific) or chondrogenesis differentiation medium (Stempro chondrogenesis differentiation kit, Gibco, Thermo Scientific) for 7 and 11 days, respectively. For adipocyte differentiation, cells were fixed with 10% formalin, washed and stained with Oil Red O for 7 min at room temperature. Cells in chondrogenic medium were fixed in ethanol, followed by Alcian blue staining. The cells were washed in phosphate-buffered saline (PBS) and analyzed by light microscopy.

### 4.4. Protein Extraction and Western Blot Analysis 

Cells were washed with PBS, scrapped in ice-cold PBS and centrifuged at 500× *g* for 5 min. The cell pellets were re-suspended directly in the reducing sample buffer (60 mM Tris-HCl, pH 6.8, 2% sodium dodecyl sulphate (SDS), 100 mM dithiothreitol and 0.01% Bromophenol Blue) in the presence of a complete EDTA-free protease inhibitor cocktail (Roche Diagnostics, Meylan, France). Proteins were separated on an SDS-polyacrylamide gel and electrotransferred to polyvinylidene difluoride membranes (Immobilon, Millipore, Dutscher, Brumath, France). Blots were blocked for 1 h with Tris-buffered saline-0.05% Tween 20 (TBS-T) supplemented with 5% nonfat milk and incubated overnight at 4 °C with a primary antibody. Filters were then washed in TBS-T, incubated for 45 min at room temperature with appropriate secondary antibodies conjugated to horseradish peroxydase and washed again prior to detection of signal with ECL plus chemilumiscent detection kit (Thermo Scientific). Primary antibodies used in this study were mouse monoclonal anti-LC3 antibody (MBL, Clinisciences, Nanterre, France) and mouse monoclonal anti-β-actin antibody (clone AC-15, Sigma-Aldrich). Original blots can be found at Figure S5.

### 4.5. Transmission Electron Microscopy

Cells were fixed in 1.6% glutaraldehyde in 0.1 M phosphate immediately after medium removal. Samples were rinsed with the same buffer and then post-fixed in osmium tetroxide (1%) for 1 h. After rinsing with distilled water, they were then dehydrated through an increasing ethanol series and embedded in epoxy resin. Ultrathin sections (70 nm) were collected on Formvar coated copper grids, stained with uranyl acetate and lead citrate and examined with a Jeol JEM 1400 transmission electron microscope equipped with a SIS Morada Camera. The number of autophagic vesicles was counted in 10–20 individual cells in each condition.

### 4.6. Effect of Thioridazine on CSC Formation

After sphere dissociation, the cells were seeded in a 24-well microplate (1000 cells/cm^2^) in the presence or the absence of thioridazine (TZ; Bio-techne, Lille, France) at 10 µM. On day 5, the spheres were counted and their diameter was measured using the AxioVision software (Zeiss). 

### 4.7. Cytotoxicity Measurement after Treatment with Digoxin and TZ

Preliminary experiments were performed to determine the optimal Digoxin concentrations to use in both CSC-enriched populations. For the analysis in spheres, the cells were treated for 1 h at 37°C with Digoxin (Sigma-Aldrich) at 5 nM. TZ (8 µM) was then added for 48 h at 37 °C. For the analysis after sphere dissociation, the cells were treated for 1 h at 37 °C with Digoxin (Sigma-Aldrich) at 5 nM for CSC-UMR and 2.5 nM for CSC-MN. TZ (30 µM) was then added for 3 h at 37 °C. Live/Dead® Viability/cytotoxicity Kit for mammalian cells (Molecular probes, Thermofisher Scientific, France) was used to determine the percentage of cell death in the absence and in the presence of Digoxin. Cells were incubated for 30 min at room temperature with Live/Dead solution (PBS, 0.5 µM Calcein-AM, 1 µM Ethidium homodimer-1). Cells were observed using a fluorescent microscope (Axiovert, Zeiss). Between 500 to 600 cells were counted for each condition (seven random fields for each slide) in two independent experiments.

### 4.8. Statistical Analysis

The results were expressed as mean ± SEM and comparisons were performed using the Student t-test. *p* values less than 0.05 were considered significant. 

## 5. Conclusions

In this work, we showed that autophagy is a critical process in OS CSCs, thus adding to the numerous resistance mechanisms they have developed. Thus, autophagy could also represent the Achille’s heel of CSCs as compounds, such as TZ, are able to switch this pathway towards cell death and could become a new therapeutic option to eradicate these cells.

## Supplementary Materials

The following are available online at https://www.mdpi.com/2072-6694/12/12/3675/s1, Figure S1: Differentiation capabilities of the parental MN and UMR cells towards adipogenic and chondrogenic lineages, Figure S2: TEM analysis of the MN cell line and the corresponding spheres in control and starvation conditions, Figure S3: Comparison of the mortality rate in UMR and MN cell lines after 16 h in HBSS, Figure S4: TEM analysis of the UMR cell line and the corresponding spheres in control and starvation conditions, Figure S5: Original WB.

## Abbreviations

ATG	Autophagy-related gene
Baf	Bafilomycin-A1
CSCs	Cancer stem cells
DMEM	Dulbecco’s modified Eagle medium
EGF	Epidermal growth factor
ER	Endoplasmic reticulum
bFGF	Basic fibroblast growth factor
HBSS	Hank’s balanced salt solution
LC3	Microtubule-associated protein 1 light chain 3 protein
MNNG/HOS	MN
MSC	Mesenchymal stem cell
mTOR	Mammalian target of rapamycin
OS	Osteosarcoma
PTEN	Phosphatase and tensin homolog
RB1	Retinoblastoma associated protein 1
TEM	Transmission electron microscopy
TZ	Thioridazine
UMR	UMR-106
