# Supplementary material for: Autophagy in Osteosarcoma Cancer Stem Cells Is a Critical Process which Can Be Targeted by the Antipsychotic Drug Thioridazine
Source: Cancers (Basel). 2020 Dec 7;12(12):3675. doi: 10.3390/cancers12123675 (PMC7762415; doi:10.3390/cancers12123675)
Supplement: Supplementary file 1 [file cancers-12-03675-s001.pdf]

Supplementary Materials:

Autophagy in Osteosarcoma Cancer Stem Cells Is a Critical Process Which Can Be Targeted by the Antipsychotic Drug Thioridazine

Olivier Camuzard, Marie-Charlotte Trojani, Sabine Santucci-Darmanin, Sophie Pagnotta, Véronique Breuil, Georges F. Carle and Valérie Pierrefite-Carle

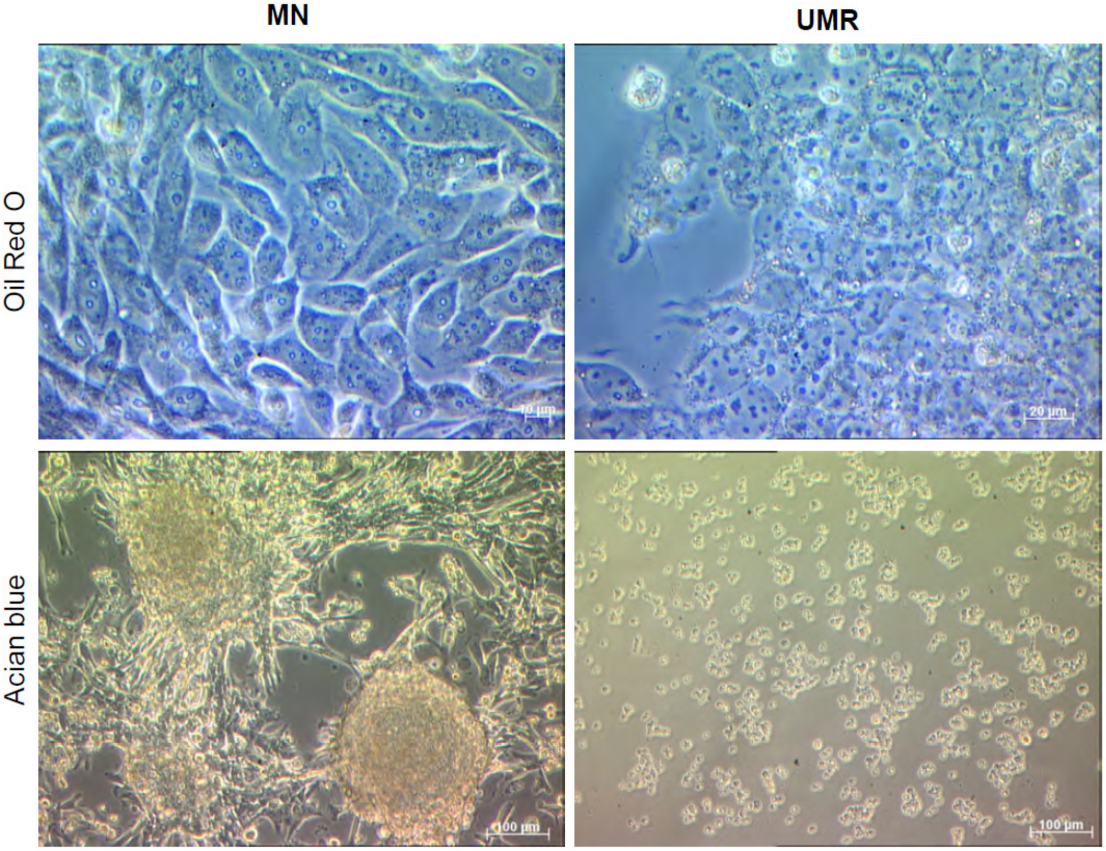

Figure S1. Differentiation capabilities of the parental MN and UMR cells towards adipogenic and chondrogenic lineages.

A

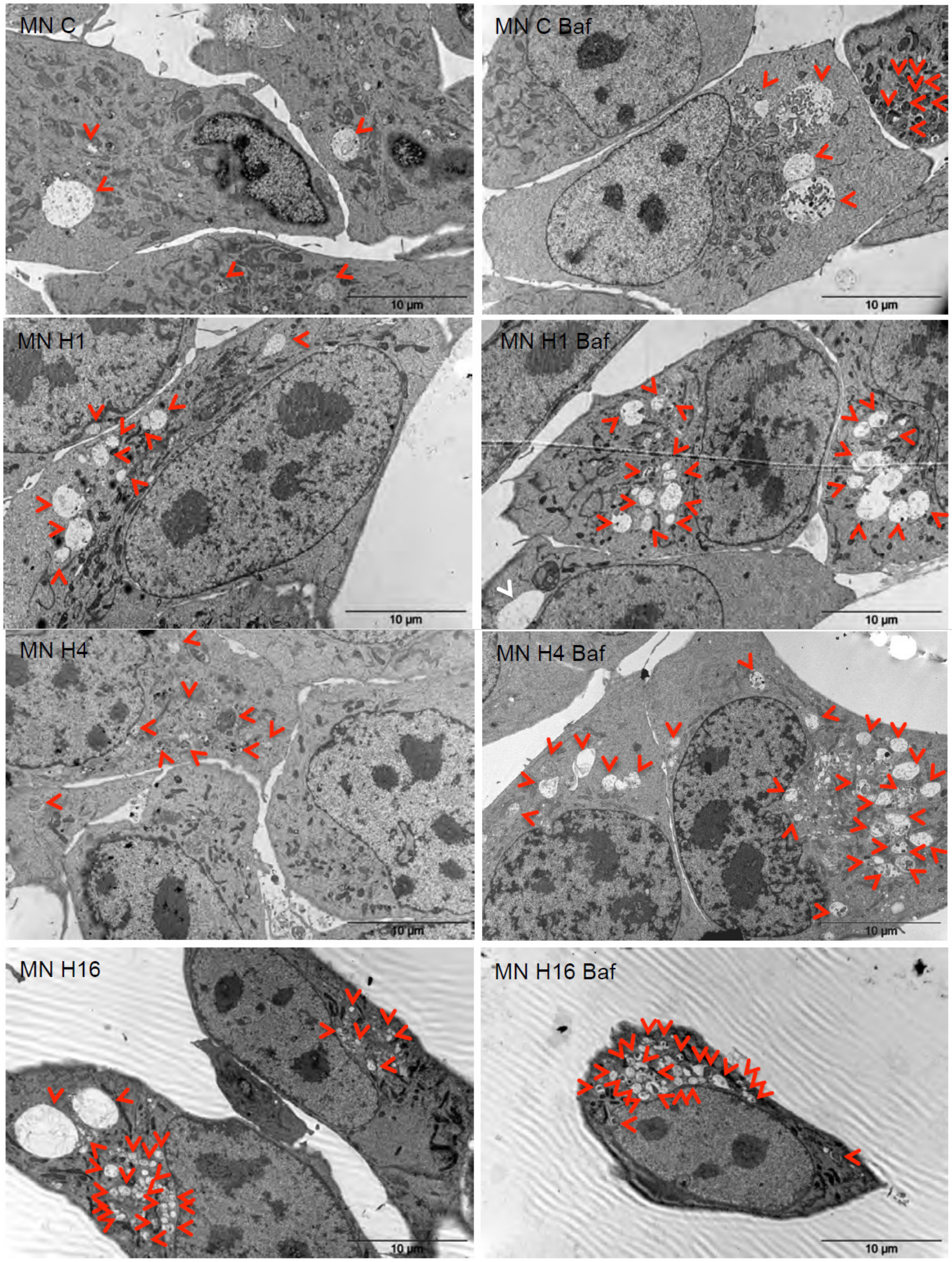
MN C
10 µm
MN C Baf
10 µm
MN H1
10 µm
MN H1 Baf
10 µm
MN H4
10 µm
MN H4 Baf
10 µm
MN H16
10 µm
MN H16 Baf
10 µm

B

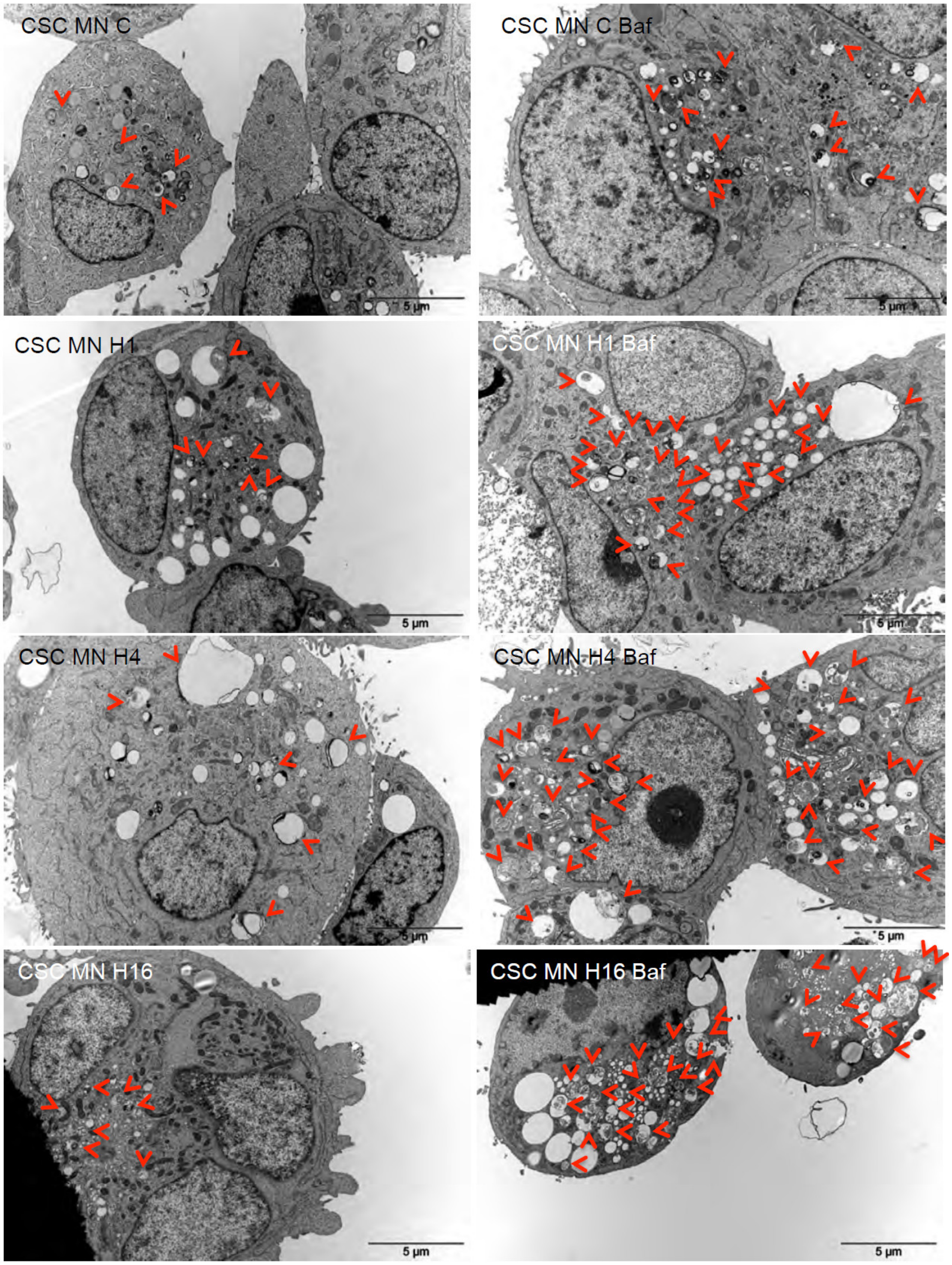

Figure S2. TEM analysis of the MN cell line and the corresponding spheres in control and starvation conditions.

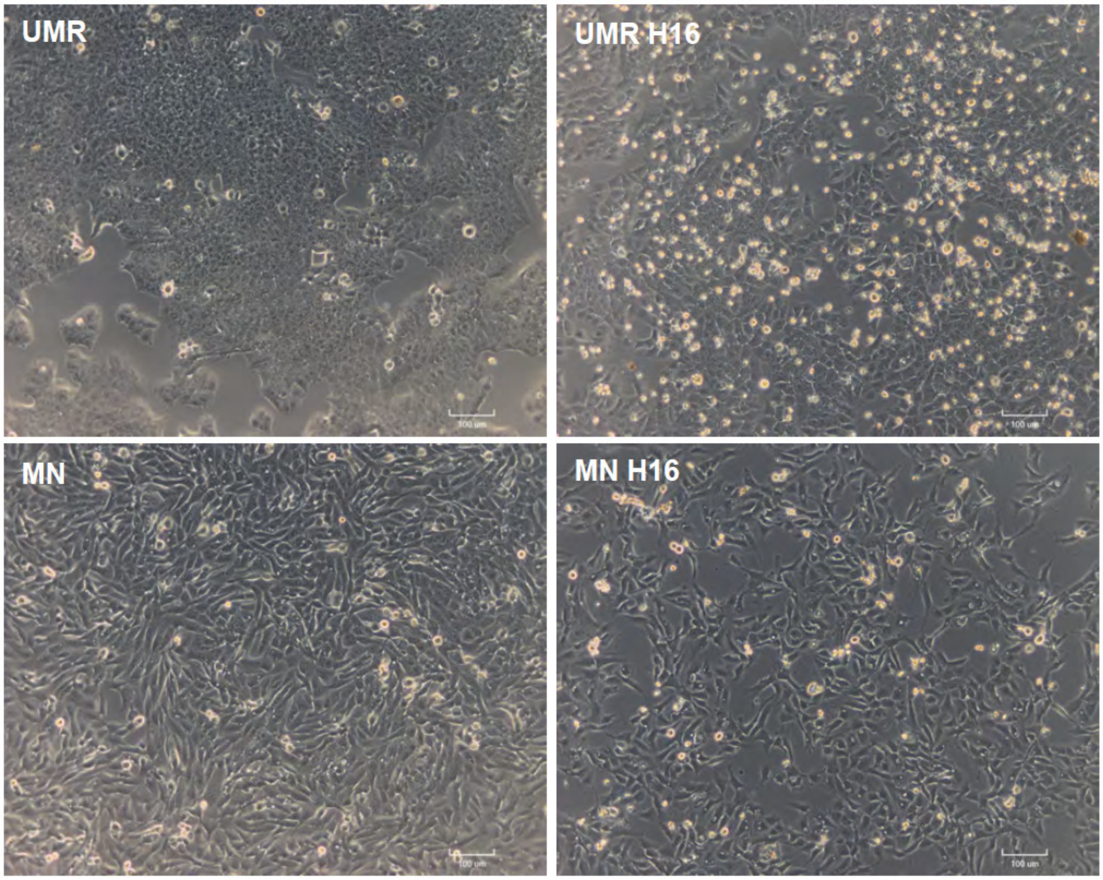

Figure S3. Comparison of the mortality rate in UMR and MN cell lines after 16 h in HBSS.

A

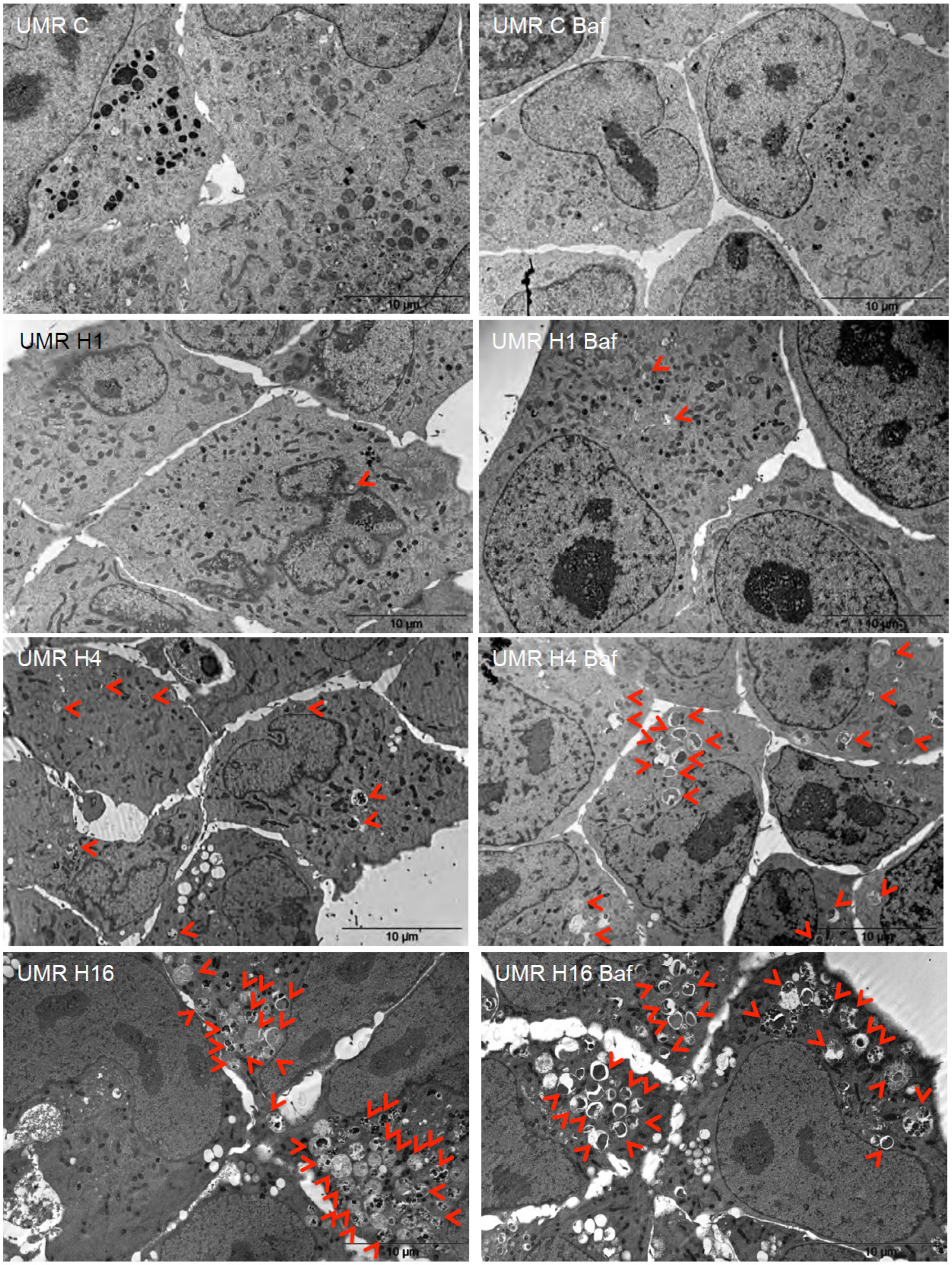
UMR C
10 µm
UMR C Baf
10 µm
UMR H1
10 µm
UMR H1 Baf
10 µm
UMR H4
10 µm
UMR H4 Baf
10 µm
UMR H16
10 µm
UMR H16 Baf
10 µm

B

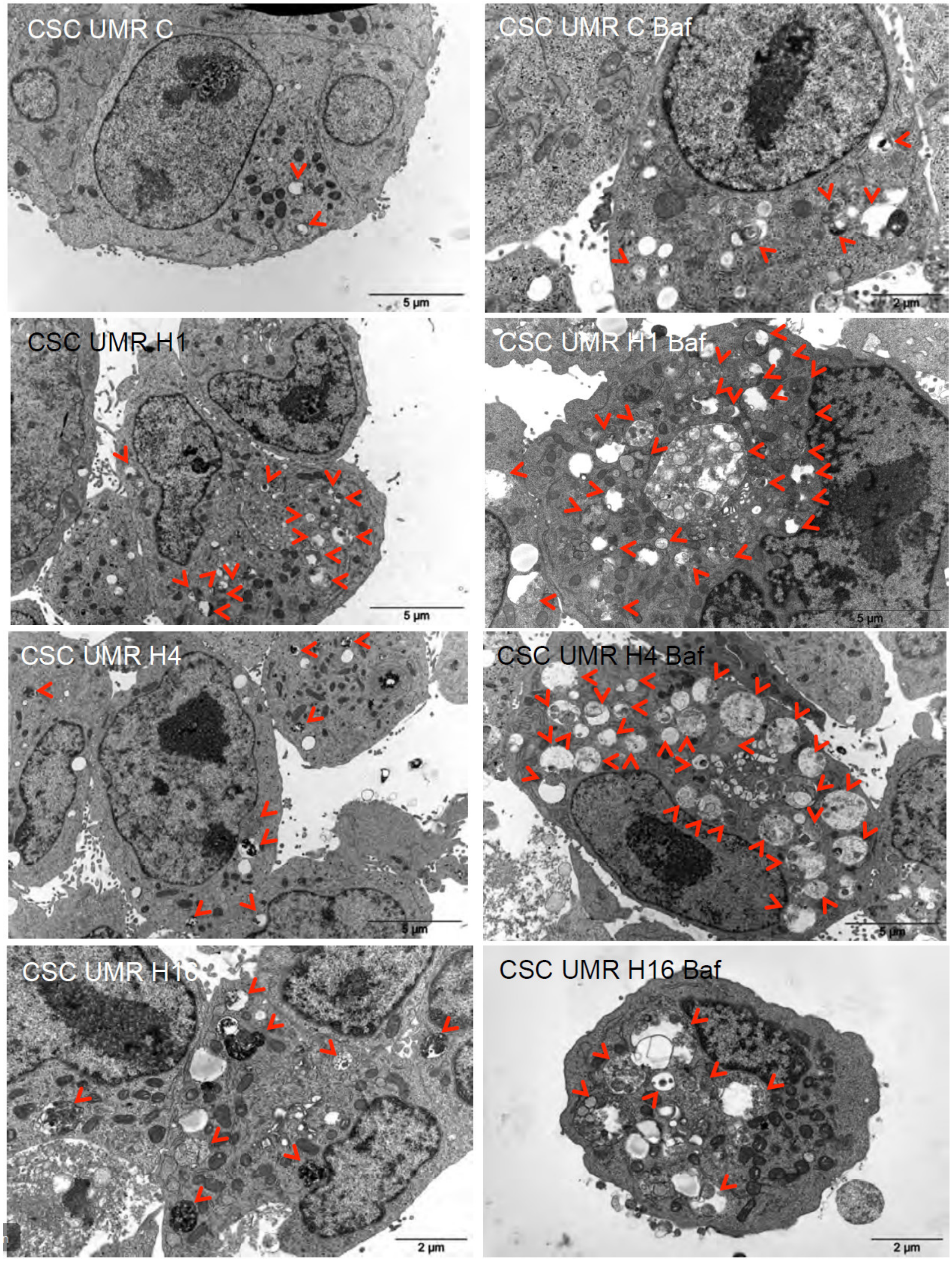

Figure S4. TEM analysis of the UMR cell line and the corresponding spheres in control and starvation conditions

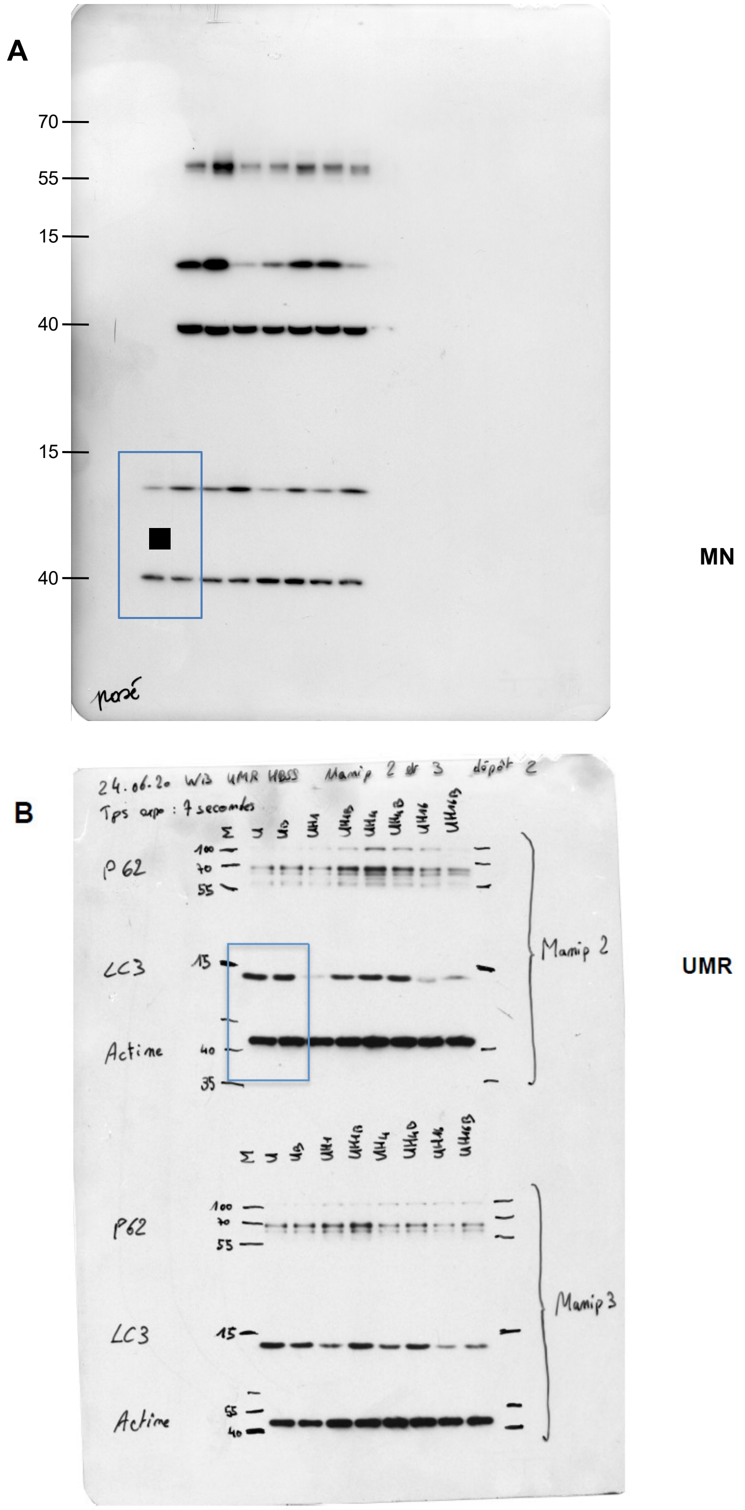

Whole blot for Figure 1

A

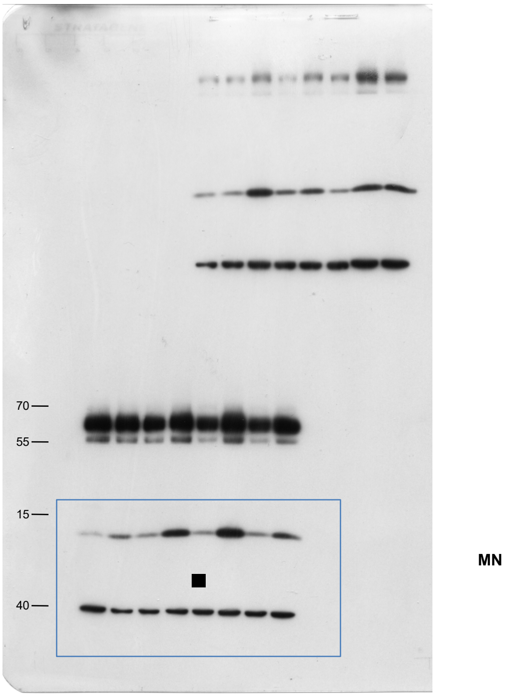

B

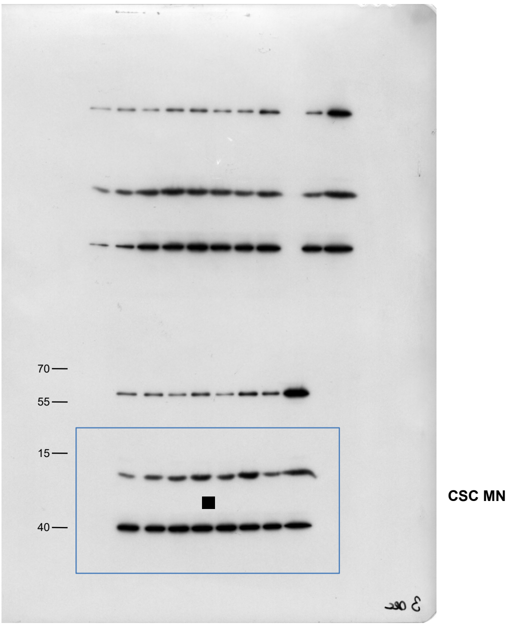

Whole blot for Figure 3

A

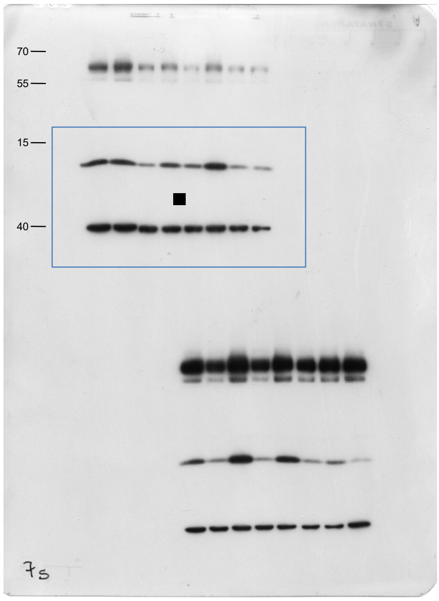

UMR

B

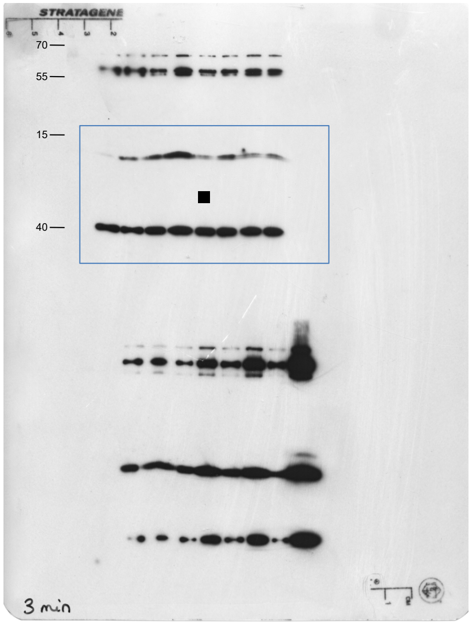

CSC UMR

Whole blot for Figure 4

A

CSC-MN

TZ 1h

TZ 5h

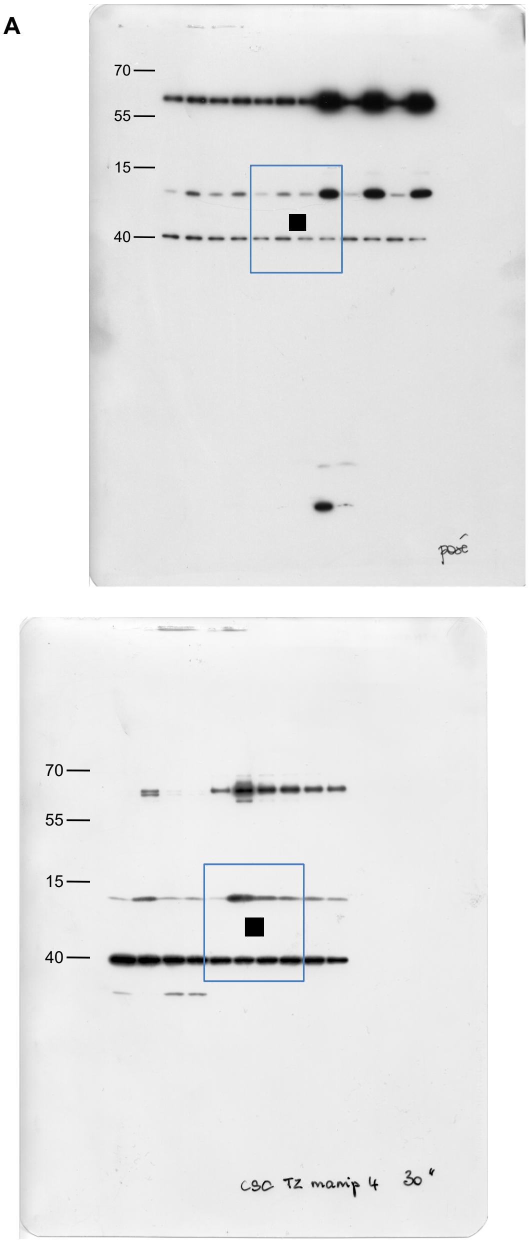

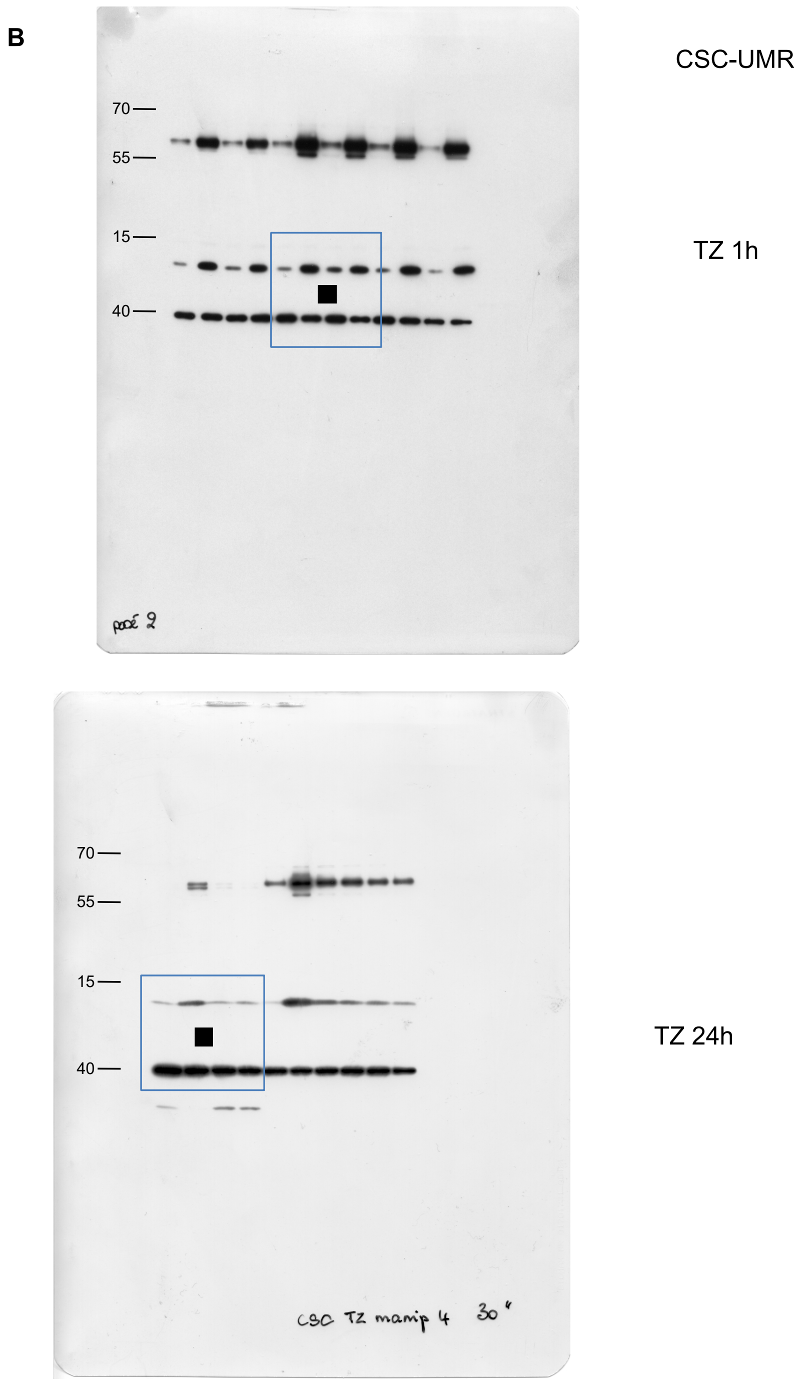

Whole blot for Figure 5

Figure S5. Original WB.

© 2020 by the authors. Licensee MDPI, Basel, Switzerland. This article is an open access article distributed under the terms and conditions of the Creative Commons Attribution (CC BY) license (http://creativecommons.org/licenses/by/4.0/).
